# Ozone therapy in femoral head necrosis: a perspective on biomarker-guided treatment rationalization and mechanistic exploration

**DOI:** 10.3389/fmed.2026.1780893

**Published:** 2026-03-17

**Authors:** Liang Zhang, Qing-hua Wang, Xu-ting Zhao, Yu-mei Ding, Xiao-dong Wang, Zhi-feng Zhang, Mu-han Na, Yi-fan Zhao, Yi Qiu, Liang-liang He

**Affiliations:** 1 Department of Anaesthesiology, The Second Affiliated Hospital of Inner Mongolia Medical University, Hohhot, China; 2 Department of Anesthesia and Perioperative Care, University of California, San Francisco, San Francisco, CA, United States; 3 Department of Anaesthesiology, People’s Hospital of Ordos Dongsheng District, Ordos, China; 4 Department of Medical Imaging, The Second Affiliated Hospital of Inner Mongolia Medical University, Hohhot, China; 5 Department of Joint Surgery, The Second Affiliated Hospital of Inner Mongolia Medical University, Hohhot, China; 6 Department of Pediatric Orthopedics, The Second Affiliated Hospital of Inner Mongolia Medical University, Hohhot, China; 7 School of Public Health, Inner Mongolia Medical University, Hohhot, China; 8 Department of Pain Management, Xuanwu Hospital Capital Medical University, National Medical Center for Neurological Diseases, National Clinical Research Center for Geriatric Diseases, Beijing, China

**Keywords:** biomarkers, bone remodeling, femoral head necrosis, oxidative stress, ozone therapy, personalized medicine, precision medicine, translational research

## Abstract

Femoral head necrosis (FHN) is a progressive and disabling disorder caused by compromised blood supply to the femoral head. Current treatments primarily aim to slow progression. However, they often fail to reverse early-stage necrosis or reliably prevent disease advancement. Importantly, most approaches lack precise molecular targets for early intervention. Ozone therapy has emerged as a potential option because of its pleiotropic biological effects, such as modulating oxidative stress, improving microcirculation, and exerting anti-inflammatory activity. However, its mechanisms remain poorly understood. Clinical evidence is limited and inconsistent, and the absence of standardized protocols hinders evidence-based implementation. To address these gaps, this perspective proposes biomarkers as a translational bridge linking ozone-induced biological responses to FHN pathology. We further outline a dynamic, biomarker-guided framework through which ozone therapy can evolve from an empirical intervention into a mechanism-informed, personalized, and more standardized precision strategy. This approach offers a scientifically grounded pathway for advancing the clinical management of FHN.

## 1 Introduction

### 1.1 Femoral head necrosis: pathophysiology and therapeutic challenges

Femoral head necrosis (FHN) is a progressive and disabling condition that most commonly results from impaired blood supply to the femoral head (1). Its pathophysiology reflects a self-perpetuating, multifactorial cascade. Initial insults—such as corticosteroid use, alcohol abuse, or trauma—may injure the vascular endothelium or promote fat embolization. These events compromise microcirculation and produce localized ischemia within the femoral head (2). The resulting hypoxic milieu induces apoptosis of osteocytes and marrow stromal cells. It also precipitates substantial oxidative stress (3). Excess reactive oxygen species damage cellular components, proteins, and deoxyribonucleic acid. This injury initiates a sustained inflammatory response. In turn, inflammation disrupts physiological bone repair and angiogenesis, thereby aggravating tissue compromise. Ultimately, these processes contribute to subchondral bone collapse and secondary osteoarthritis (4). Current hip-preservation strategies, including core decompression and bone grafting, can delay progression in selected patients. However, they often do not reverse early-stage necrosis and may not reliably prevent its advancement (5, 6). Moreover, these approaches lack precise molecular targets that would enable earlier and more mechanism-directed treatment.

### 1.2 Ozone therapy: a multi-target biological intervention

Ozone therapy has attracted attention as a potential multi-modal intervention because of its diverse biological actions. Mechanistically, its effects are largely mediated by reactive oxygen species and lipid oxidation products generated when ozone interacts with biological fluids (7). At controlled doses, these products can act as mild oxidative stimuli. They activate endogenous antioxidant defenses, particularly the nuclear factor erythroid 2-related factor 2 (Nrf2) pathway, thereby upregulating enzymes such as superoxide dismutase and glutathione peroxidase (7, 8). This phenomenon is often described as “oxidative preconditioning,” which may enhance cellular resistance to subsequent injury (7). Ozone therapy may also improve hemorheological properties by increasing erythrocyte flexibility and reducing platelet aggregation. These effects could support perfusion in ischemic tissues (9). In addition, ozone exhibits anti-inflammatory and immunomodulatory actions. For example, it can modulate nuclear factor kappa-light-chain-enhancer of activated B cells activity and reduce the expression of pro-inflammatory cytokines (10, 11). These properties provide a mechanistic rationale for evaluating ozone therapy in FHN, where oxidative stress, microcirculatory impairment, and inflammation are central drivers of disease progression. Clinically, ozone has been applied in orthopedics and pain management with reported benefits. However, the overall evidence base remains preliminary (11, 12).

### 1.3 Unresolved issues: from empirical use to mechanism-driven therapy

Although some clinical reports suggest symptomatic improvement in FHN following ozone therapy, the evidence remains inconsistent and methodologically limited. Substantial heterogeneity exists in ozone concentration, dosage, administration route, and treatment schedule. This variability reduces comparability across studies and complicates interpretations of outcomes. Two major scientific gaps currently limit evidence-based adoption. First, ozone’s mechanism of action within the necrotic bone microenvironment remains insufficiently defined. Specifically, it is unclear how ozone modulates the interplay among ischemia, oxidative stress, apoptosis, and impaired repair processes in FHN. Second, validated biomarkers are lacking for objective response monitoring and for identifying patients most likely to benefit. Current assessments rely heavily on delayed imaging changes and subjective symptom reports. These approaches are not optimal for real-time evaluation of biological responses (13). Therefore, advancing ozone therapy beyond empirical use will require mechanistic clarification and biomarker validation. Such progress would support a more targeted, personalized, and standardized therapeutic strategy for FHN.

## 2 Biomarkers: bridging ozone therapy and FHN pathology

**Figure 1:**
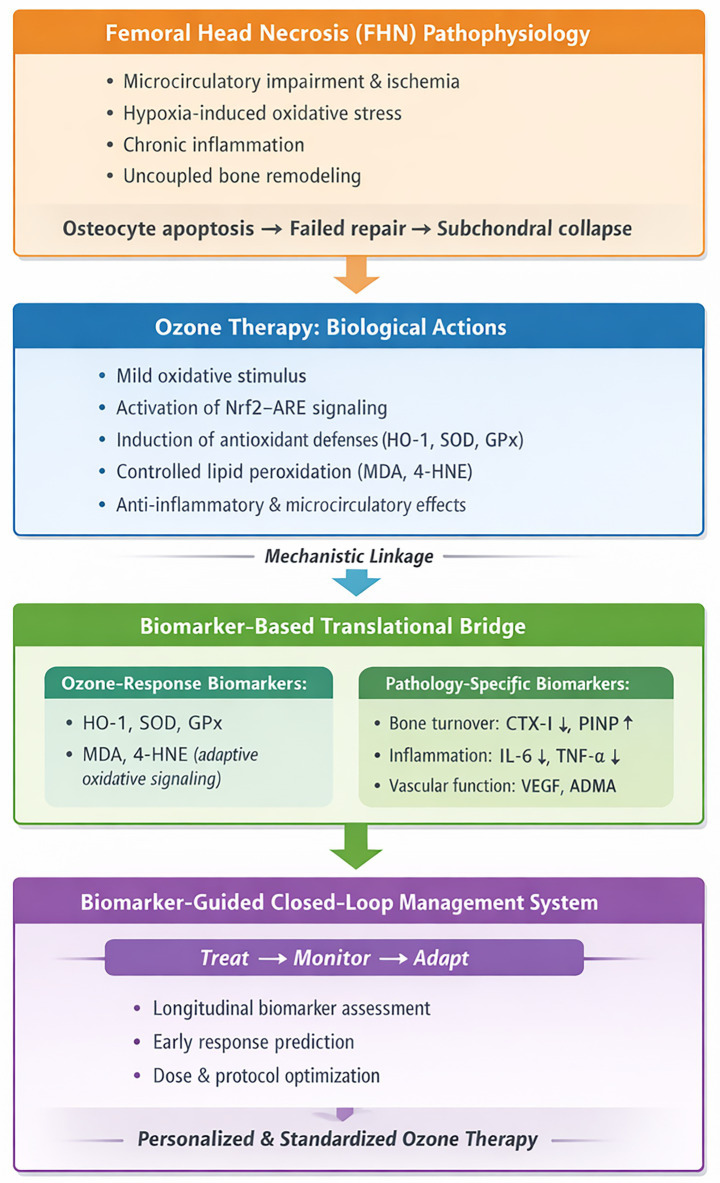
Biomarker-guided translational framework linking ozone therapy to femoral head necrosis pathology.

Biomarkers provide a structured framework for elucidating and optimizing ozone therapy in FHN (Figure 1). As objective and quantifiable indicators, biomarkers can inform mechanisms of action, improve risk stratification, predict outcomes, and support individualized treatment decisions.

### 2.1 Biomarkers of FHN pathology

Pathology-oriented biomarkers reflect key biological processes across FHN stages. Bone turnover markers—such as increased C-terminal telopeptide of type I collagen (CTX-I) and reduced procollagen type I N-terminal propeptide (PINP)—may capture uncoupled remodeling during disease progression (14, 15). Biomarkers of vascular dysfunction, including asymmetric dimethylarginine and altered vascular endothelial growth factor levels, can reflect endothelial impairment and angiogenic capacity (16, 17). Inflammatory mediators, such as interleukin-6 (IL-6) and tumor necrosis factor alpha (TNF-α), indicate sustained inflammatory activation that exacerbates tissue injury and inhibits repair (18). Collectively, these biomarkers provide a multidimensional assessment of disease activity that extends beyond structural imaging.

### 2.2 Biomarkers of ozone response

Ozone-response biomarkers capture biological effects induced by treatment (Table 1). Representative molecules include heme oxygenase-1 (HO-1) and NAD(P)H quinone dehydrogenase 1. Their upregulation via the Nrf2/antioxidant response element axis indicates activation of endogenous antioxidant defenses (7, 19, 20). Enzymatic activity of superoxide dismutase (SOD) and glutathione peroxidase, measured in serum or local samples, can quantify redox modulation after ozone exposure (7, 21). In addition, lipid peroxidation products—such as malondialdehyde (MDA) and 4-hydroxynonenal—may serve as markers of controlled oxidative signaling. Importantly, these markers can help distinguish regulated oxidative responses from pathological oxidative injury when interpreted in context (7, 22). Systematic monitoring of these biomarkers can confirm target engagement and support mechanistic inference regarding ozone’s proposed therapeutic cascade.

**Table 1:** Representative biomarker categories linking femoral head necrosis pathology with ozone therapeutic response.

Biomarker category	Representative biomarkers	Pathophysiological relevance	Role in ozone-guided management
Bone remodeling	CTX-I, PINP	Reflect uncoupled bone turnover	Monitor repair normalization
Vascular function	VEGF, ADMA	Endothelial dysfunction, angiogenesis	Assess perfusion response
Inflammation	IL-6, TNF-α	Sustained inflammatory damage	Track anti-inflammatory effects
Oxidative stress	HO-1, SOD, MDA	Redox balance and adaptive response	Confirm target engagement

CTX-I, C-terminal telopeptide of type I collagen; PINP, procollagen type I N-terminal propeptide; VEGF, vascular endothelial growth factor; ADMA, asymmetric dimethylarginine; IL-6, interleukin-6; TNF-α, tumor necrosis factor alpha; HO-1, heme oxygenase-1; SOD, superoxide dismutase; MDA, malondialdehyde.

### 2.3 A bridging hypothesis for mechanism and efficacy

We propose that clinical benefit from ozone therapy in FHN depends on measurable modulation of ozone-response biomarkers. These changes should subsequently drive favorable shifts in pathology-specific biomarkers. Specifically, an effective regimen may first increase HO-1 expression, SOD activity, and transient lipid peroxidation signaling (7, 8). These early responses should then correlate with reduced inflammatory cytokines (e.g., IL-6 and TNF-α), normalization of bone turnover markers (decreased CTX-I and increased PINP), and improved vascular indicators (8, 11). This sequence—from ozone-induced signaling to downstream pathological modulation—would provide a mechanistically coherent link between treatment and disease modification (23). Ideally, biomarker improvement should precede and correlate with clinical benefits in pain, function, and imaging-based outcomes. Rigorous validation of this hypothesis will require longitudinal studies integrating synchronized biomarker, clinical, and radiological assessments.

## 3 Toward a biomarker-driven framework for research and clinical practice

To translate mechanistic hypotheses into actionable clinical strategies, a structured framework integrating longitudinal biomarker measurement with clinical endpoints is required. Such a framework can clarify mechanisms, optimize protocols, and facilitate standardization across settings.

### 3.1 Mechanistic study design

Mechanistic investigation would benefit from standardized, multicenter prospective cohorts. Protocols should incorporate systematic biospecimen collection (e.g., serum and synovial fluid) at prespecified timepoints: baseline, early treatment (e.g., after 1–2 sessions), mid-treatment, end of treatment, and follow-up (24). Multi-omics profiling can provide mechanistic depth. Proteomics may capture dynamic alterations in signaling proteins, cytokines, and enzymes linked to inflammation, oxidative stress, and bone remodeling (25). Metabolomics can identify shifts in energy metabolism and lipid peroxidation pathways, offering functional insight into treatment responses (26). Targeted assays should quantify representative markers from ozone-response (e.g., HO-1 and MDA) and pathology-related categories (e.g., CTX-I and IL-6) (7, 8, 11, 27). These molecular data should be analyzed alongside serial clinical metrics (pain and functional scores) and quantitative imaging parameters (e.g., magnetic resonance imaging-derived indices). Together, such an approach could generate a temporal molecular map of ozone therapy in FHN and identify mediators associated with clinical benefit.

### 3.2 Predictive and monitoring models

Longitudinal biomarker trajectories may support predictive and monitoring models. Machine-learning approaches applied to baseline multi-omics profiles could identify clinically meaningful endotypes. For example, one subtype may exhibit high inflammatory activation (elevated IL-6/TNF-α), whereas another may show metabolic dysregulation reflected by lipid signatures (27, 28). Early biomarker dynamics (e.g., the magnitude of HO-1 induction or early IL-6 decline) may function as time-sensitive predictors of long-term structural and functional outcomes (29). A favorable early signature could support treatment continuation, whereas absent or unfavorable biomarker shifts may justify earlier reassessment (30). This strategy could shift clinical decision-making from uniform application toward stratified, precision-oriented management.

### 3.3 Personalization and standardization via a closed-loop system

These insights could be operationalized through a biomarker-guided closed-loop management model structured around a “Treat–Monitor–Adapt” cycle. After initiating a standardized ozone protocol, patients would undergo scheduled monitoring using a defined biomarker panel (31). If monitoring indicates insufficient antioxidant activation or persistent inflammatory activity, treatment parameters could be adjusted in a pre-specified manner. Adjustments might include ozone concentration, dose per session, or treatment frequency (32).

The goal of iterative adjustment would be to steer biomarker trajectories toward a desired therapeutic response signature (33). Aggregating these data across cohorts may also support evidence synthesis for consensus-driven protocol development and clinical standardization (34). In this way, biomarkers can facilitate both personalization and reproducible practice.

## 4 Challenges and future directions

### 4.1 Key challenges

Despite their promise, several challenges limit biomarker implementation in ozone therapy for FHN. First, biomarker specificity and sensitivity remain suboptimal. Many commonly used inflammatory and oxidative stress markers lack disease specificity and may be influenced by systemic comorbidities (35). Their dynamic range may also be insufficient to reliably distinguish responders from non-responders.

Second, standardization of sampling—particularly for local biomarkers (e.g., synovial fluid or tissue)—is difficult. Variability in collection sites, techniques, processing steps, and storage conditions can compromise comparability and reproducibility (36). Third, longitudinal multi-omics profiling is costly and resource-intensive, which can hinder large-scale implementation (37, 38). Finally, interpreting high-dimensional biomarker data requires robust bioinformatics and statistical strategies to integrate signals, separate correlation from causation, and establish defensible links to clinical outcomes (39).

### 4.2 Future directions

Future efforts should prioritize approaches that address these limitations. First, multicenter collaborations could establish standardized biorepositories and integrated clinical–molecular databases. This would strengthen consistency in sample handling and facilitate data sharing (40). Second, minimally invasive monitoring strategies, including liquid biopsy approaches based on circulating exosomes or cell-free nucleic acids, may reduce patient burden and enable dynamic longitudinal assessment (41). Third, combining molecular biomarkers with imaging-derived digital biomarkers—such as radiomic magnetic resonance imaging features—may improve early detection and prediction of femoral head collapse within a multimodal framework (42). Fourth, randomized controlled trials incorporating validated biomarkers as secondary or exploratory endpoints would strengthen causal inference and clarify therapeutic mechanisms (43, 44). Collectively, these advances could support evidence-based protocols and promote individualized care in FHN.

## 5 Summary

Biomarkers may serve as a critical translational bridge for advancing ozone therapy in FHN. The field should move from empirically guided supportive use toward biomarker-informed, mechanism-based, and individualized strategies. Systematic identification of ozone-responsive biomarkers, coupled with longitudinal correlation to established disease-pathology markers, may clarify mechanisms and enable objective response monitoring and efficacy prediction. Future work should prioritize biomarker-driven translational studies supported by interdisciplinary collaboration across orthopedics, immunology, molecular biology, and bioinformatics. Rigorous integration of clinical trials with mechanistic research will be essential to define ozone therapy’s clinical role in FHN and to optimize patient outcomes through more precise and evidence-based management.

## Data Availability

The original contributions presented in the study are included in the article/supplementary material, further inquiries can be directed to the corresponding authors.
